# *FoxK1* is Required for Ectodermal Cell Differentiation During Planarian Regeneration

**DOI:** 10.3389/fcell.2022.808045

**Published:** 2022-02-22

**Authors:** Pablo Coronel-Córdoba, M. Dolores Molina, Gemma Cardona, Susanna Fraguas, Eudald Pascual-Carreras, Emili Saló, Francesc Cebrià, Teresa Adell

**Affiliations:** ^1^ Department of Genetics, Microbiology and Statistics, Faculty of Biology, University of Barcelona, Barcelona, Spain; ^2^ Institute of Biomedicine of the University of Barcelona (IBUB), Barcelona, Spain

**Keywords:** FOXK, planarian, nervous system, regeneration, stem cell, differentiation

## Abstract

Forkhead box (Fox) genes belong to the “winged helix” transcription factor superfamily. The function of some Fox genes is well known, such as the role of *foxO* in controlling metabolism and longevity and *foxA* in controlling differentiation of endodermal tissues. However, the role of some Fox factors is not yet well characterized. Such is the case of FoxK genes, which are mainly studied in mammals and have been implicated in diverse processes including cell proliferation, tissue differentiation and carcinogenesis. Planarians are free-living flatworms, whose importance in biomedical research lies in their regeneration capacity. Planarians possess a wide population of pluripotent adult stem cells, called neoblasts, which allow them to regenerate any body part after injury. In a recent study, we identified three *foxK* paralogs in the genome of *Schmidtea mediterranea*. In this study, we demonstrate that *foxK1* inhibition prevents regeneration of the ectodermal tissues, including the nervous system and the epidermis. These results correlate with *foxK1* expression in neoblasts and in neural progenitors. Although the triggering of wound genes expression, polarity reestablishment and proliferation was not affected after *foxK1* silencing, the apoptotic response was decreased. Altogether, these results suggest that *foxK1* would be required for differentiation and maintenance of ectodermal tissues.

## Introduction

Forkhead box (Fox) genes belong to the “winged helix” superfamily of transcription factors, showing a Forkhead DNA-binding domain. Over 2,000 Fox proteins, phylogenetically classified into 25 families (A to S), have been identified in a number of species of fungi and metazoans (Benayoun, Caburet, and Veitia 2011; Pascual-Carreras et al., 2021). Fox genes control essential processes such as cell death, cell cycle and cell differentiation during all stages of development and in adult tissues, although many members show a tissue/stage-specific expression and function (Benayoun, Caburet, and Veitia 2011). The function and the implication of some Fox genes in human diseases is well known, such as the role in the differentiation of endodermal tissues of FoxA factors, the control of the cell cycle by FoxM, the role of FoxO in regulating metabolism and longevity and the contribution to speech acquisition of FoxP (Golson and Kaestner 2016). Some Fox transcription factors, especially FoxA, FoxO and FoxI members, are shown to act as pioneer factors, which open compacted chromatin to facilitate the binding of other transcription factors at enhancer sites (Lalmansingh, 2012). However, the function of some Fox families has not been properly addressed. An example are the FoxK genes, which are characterized by containing a forkhead-associated domain (FHA) besides the Forkhead DNA-binding domain, which mediates its interaction with multiple proteins (Ji et al., 2021). Few studies have been reported on the function of *foxK* genes, and they are mainly performed in mammals, which show two members (FoxK1 and FoxK2). From these studies, it is known that FoxK proteins are not tissue-specific but are ubiquitously expressed, and they regulate diverse biological processes, including cell proliferation, myogenic differentiation, cell cycle, autophagy, DNA damage and carcinogenesis (Liu et al., 2019).

Planarians are Lophotrochozoans well known for possessing pluripotent adult stem cells (called neoblasts) that confer them an extreme plasticity. They can regenerate any body part and change their size according to food availability (Wagner, Wang, and Reddien 2011; Baguñà 2012; Cebrià, Adell, and Saló 2018; Molina and Cebrià 2021). Previous studies of Fox genes function in planarians have demonstrated their evolutionary conserved role, and provided new data about their cellular function (Scimone, Lapan, and Reddien 2014; Vogg et al., 2014; He et al., 2017; Scimone et al., 2018). For instance, *foxA* was found to be essential for the maintenance of the pharynx and endodermal tissues (Koinuma et al., 2000; Adler et al., 2014) and *foxO* showed a conserved role in regulating metabolism during planarian regeneration and homeostasis (Pascual-Carreras et al., 2021). In a recent study, we identified 3 *foxK* paralogs in the genome of the planarian species *S. mediterranea* (*Smed foxK-1-2-3*). In this study, we aimed to analyze the function of *Smed-Fox* genes to provide new data about the role of this family in a model that, in contrast to mammals, displays stem cell-based tissue maintenance and regeneration.

We found that *foxK1*, expressed in neoblasts and their progenitors, is required for regeneration of neural and epidermal tissues. We hypothesize that *foxK1* may play a role in activating and maintaining lineage specific enhancers.

## Results

### *foxK1* RNAi Planarians Can Properly Regenerate Neither the Eyes nor the Nervous System

**FIGURE 1:**
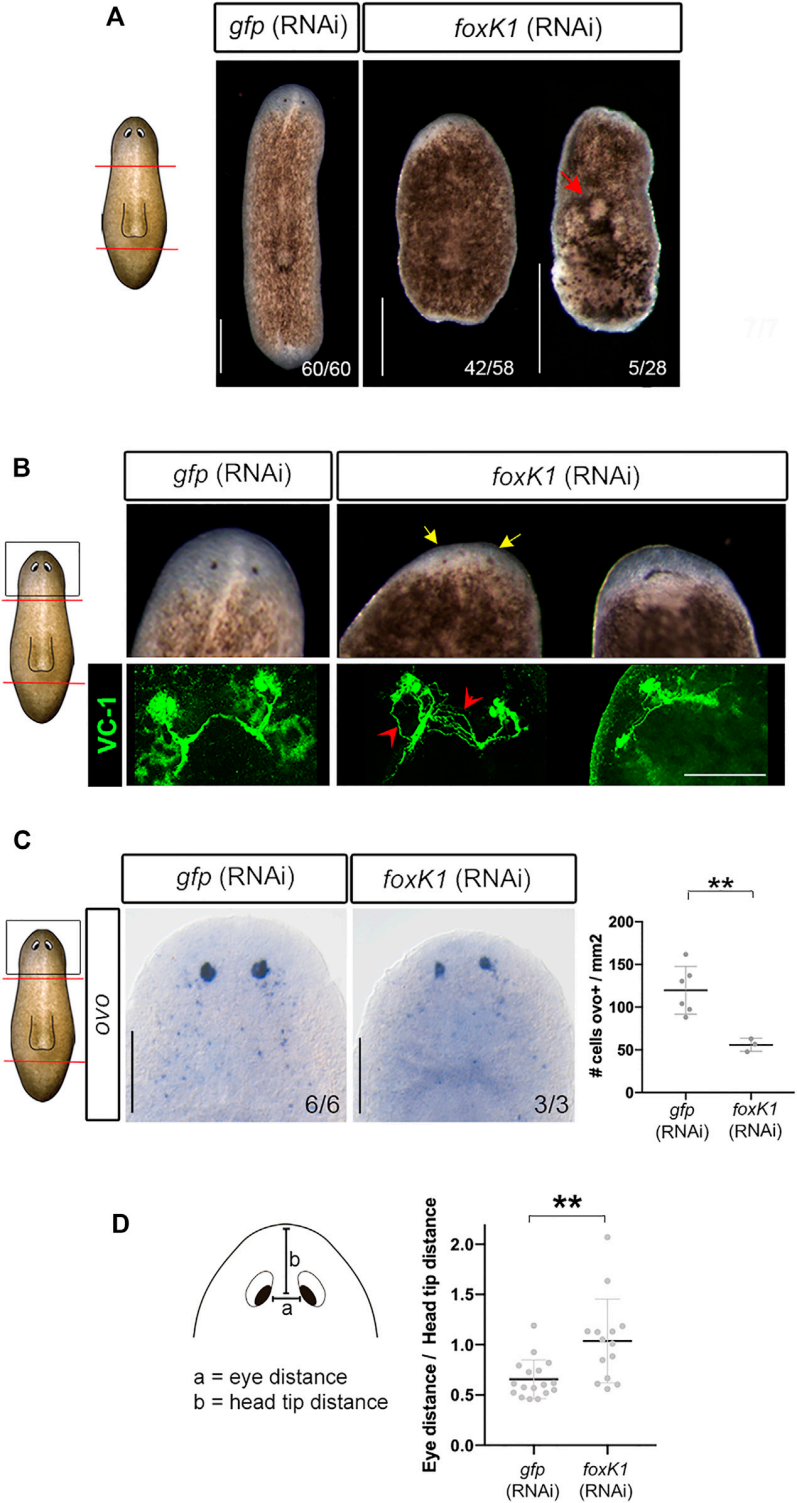
*foxK1* silencing impairs planarian regeneration and eye differentiation. **(A)** *foxK1* RNAi animals present smaller anterior and posterior blastemas in comparison to controls. Red arrowhead points to the epidermal injuries observed in *foxK1* RNAi animals. Scale bar: 500 µm. **(B–D)** Aberrant eye regeneration after silencing *foxK1*. **(B)** *foxK1* RNAi animals present smaller eyes that differentiate closer to the pre-existing tissue in comparison to controls (yellow arrows). Visualization of the eyes with an anti-VC1 immunostaining reveals defects in eye regeneration and an incorrect guidance of visual axons (red arrows). Scale bar: 100 µm. **(C)** Whole-mount *in situ* hybridization analysis of the expression of *ovo* in *foxK1* RNAi animals. Scale bar: 200 µm. Quantification of *ovo* + cells/mm2 shows a significant decrease in the number of eye progenitor cells in *foxK1* RNAi animals (*n* = 3) compared to *gfp* RNAi animals (*n* = 6). Values represent the mean ± standard deviation [s.d]. **p-value < 0.01. **(D)** Quantification of the eye distance/head tip distance ratio shows a significant increase in *foxK1* RNAi animals (*n* = 14) compared to control *gfp* (RNAi) animals (*n* = 17). **p-value < 0.01. **(A–D)**: All animals shown and analyzed are at 7 days of regeneration after anterior-posterior amputation. In the schematic planarians, red lines indicate the amputation planes and black squares the region analysed.

In a previous study, we identified 3 paralogs within the FoxK family in *S. mediterranea* (*foxK1-2-3*) (Pascual-Carreras et al., 2021). In this study, we have inhibited the three *foxK* genes by RNAi, showing that RNAi of *foxk1* generates the strongest phenotype (Figure 1A) (Supplementary Figure S1A). *foxk1* (RNAi) animals cannot regenerate proper anterior or posterior structures and 25% of them die after developing epidermal lesions after a single round of injection and amputation (Figure 1A). Prolonged silencing of *foxK1* eventually impaired the viability of all the treated animals.

*FoxK1* RNAi planarians regenerated smaller blastemas and eyes (Figure 1A). In some animals, eyes were merged in the midline (Figure 1B). Immunostaining of photoreceptors demonstrated that visual axons were aberrant and not properly connected to form a correct optic chiasm (Figure 1B). The expression of *ovo*, a pan-eye marker (Lapan and Reddien 2011), corroborated the decrease in the number of both differentiated and progenitor eye cells (Figure 1C). It also showed that eyes were more separated and closer to the anterior tip in *foxK1*-silenced planarians (Figure 1D). A phototaxis assay showed that *foxK1* RNAi animals do not respond to the light stimulus, suggesting that although they could regenerate some photoreceptor cells (Figure 1B), these were not fully functional (Supplementary Figure S1B).

**FIGURE 2:**
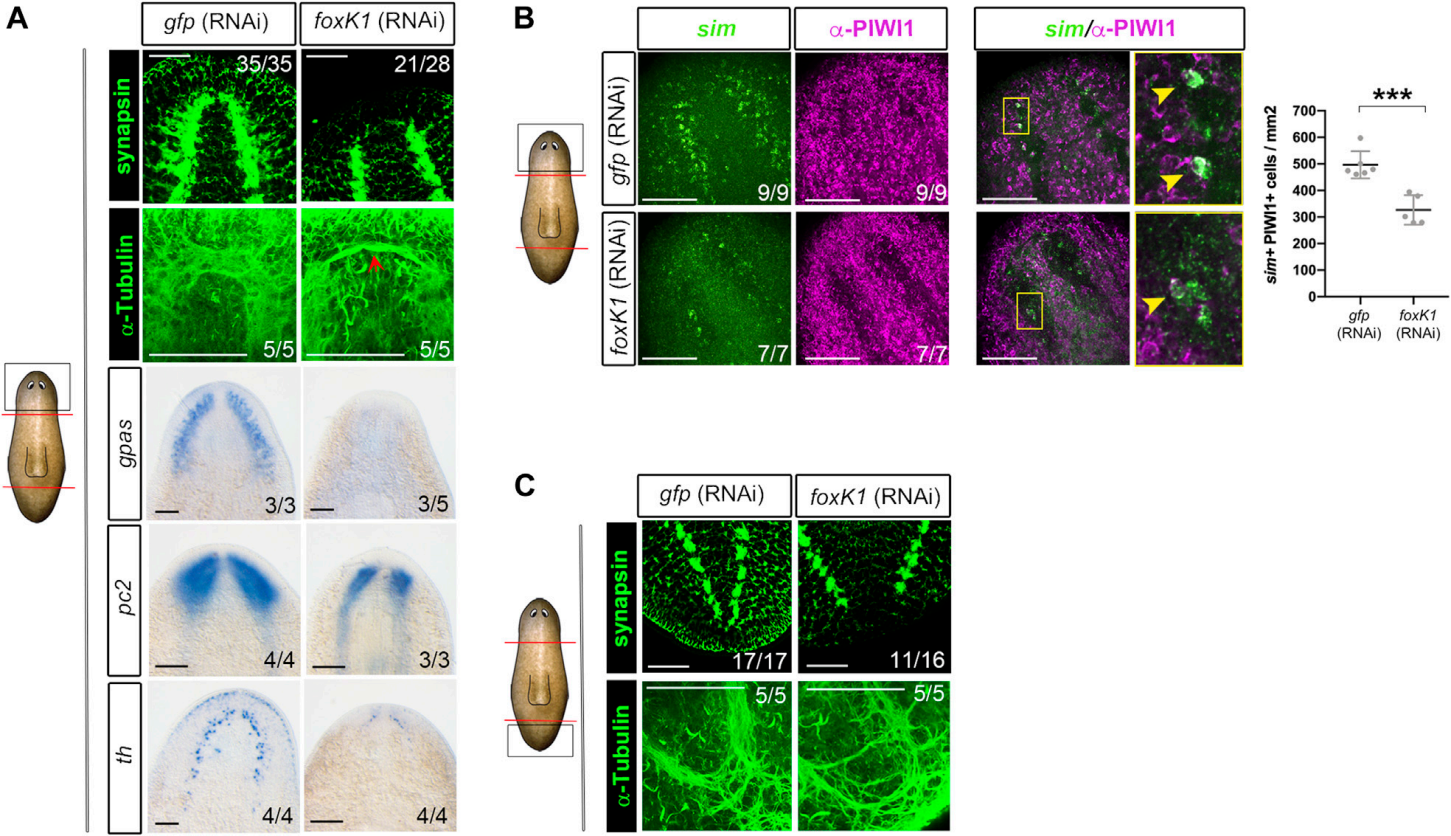
*foxK1* silenced animals show a reduced number of neural progenitors and almost absent differentiated neural structures. **(A)** Whole-mount immunostaining and *in situ* hybridization analyses (WISH) of the anterior nervous system regeneration in *foxK1* RNAi animals 7 days post amputation. From top to bottom: immunostaining of the brain ganglia visualized with anti-synapsin which labels the brain synapsis; Immunostaining of the brain ganglia visualized with anti-*α*-tubulin which labels axons; WISH of *gpas, pc2 and th* which label differentiated neural cells. Scale bar: 100 µm. **(B)** Whole-mount fluorescence *in situ* hybridization analysis (WFISH) combined with immunostaining of the anterior blastemas in *foxK1* RNAi animals 7 days post amputation. From left to right: WFISH with *sim,* which labels both neural progenitors and differentiated neurons; immunostaining with anti-PIWI1 which labels stem cells; double *sim+/*PIWI1+ positive cells, which indicate neural progenitors; Scale bar: 100 µm. Quantification of *sim +* PIWI + cells/mm2 reveals a decreased number of neural progenitors in *foxK1* RNAi animals. Values represent the mean ± standard deviation [s.d] of a mean of 5 samples per condition. ***p-value < 0.001. **(C)** Whole-mount immunostaining analysis of the posterior nervous system regeneration in *foxK1* RNAi animals at 7 days post amputation. From top to bottom: anti-synapsin labelling the synapsis and anti-*α*-tubulin labeling the axons, showing defects in the posterior VNCs. Scale bar: 100 µm. In the schematic planarians, red lines indicate the amputation planes and black squares the region analysed.

Analysis of the nervous system of *foxK1* RNAi animals through anti-synapsin and anti-*α*-tubulin immunostaining showed that cephalic ganglia did not regenerate, and only a few axons at the level of the neural nerve cords (VNCs) crossed the midline (Figure 2A). The analysis of the expression of markers of differentiated neural cells such as *gpas* (Cebrià, Nakazawa, and Mineta 2002; Iglesias et al., 2011), *pc2* (Collins et al., 2010) and *th* (Nishimura et al., 2007; Fraguas et al., 2011) corroborated that cephalic ganglia could not be formed (Figure 2A). The expression of *sim*, a marker of differentiated and progenitor neural cells (Cowles et al., 2013), was decreased in *foxK1* RNAi animals (Supplementary Figure S2A). Quantification of differentiated (*sim+*) and progenitor (*sim+*/PIWI1+) neural cells indicated that both populations were decreased in *foxK1* RNAi planarians (Figure 2B). The problem in regenerating the nervous system was not anterior specific, but it also affected the regeneration of proper posterior VNCs in the tail (Figure 2C).

According to the RNAi phenotype, *foxK1* was expressed in the nervous system of intact animals and during regeneration (Supplementary Figure S1C). Furthermore, the analysis of the expression pattern of *foxK1* in irradiated animals showed that it was expressed in differentiated cells and also in progenitors (Supplementary Figure S1C), which agrees with published single-cell RNAseq databases (Plass et al., 2018; Zeng et al., 2018) (Supplementary Figures S1D–F).

### *foxK1* RNAi Animals Cannot Regenerate a Proper Epidermis

**FIGURE 3:**
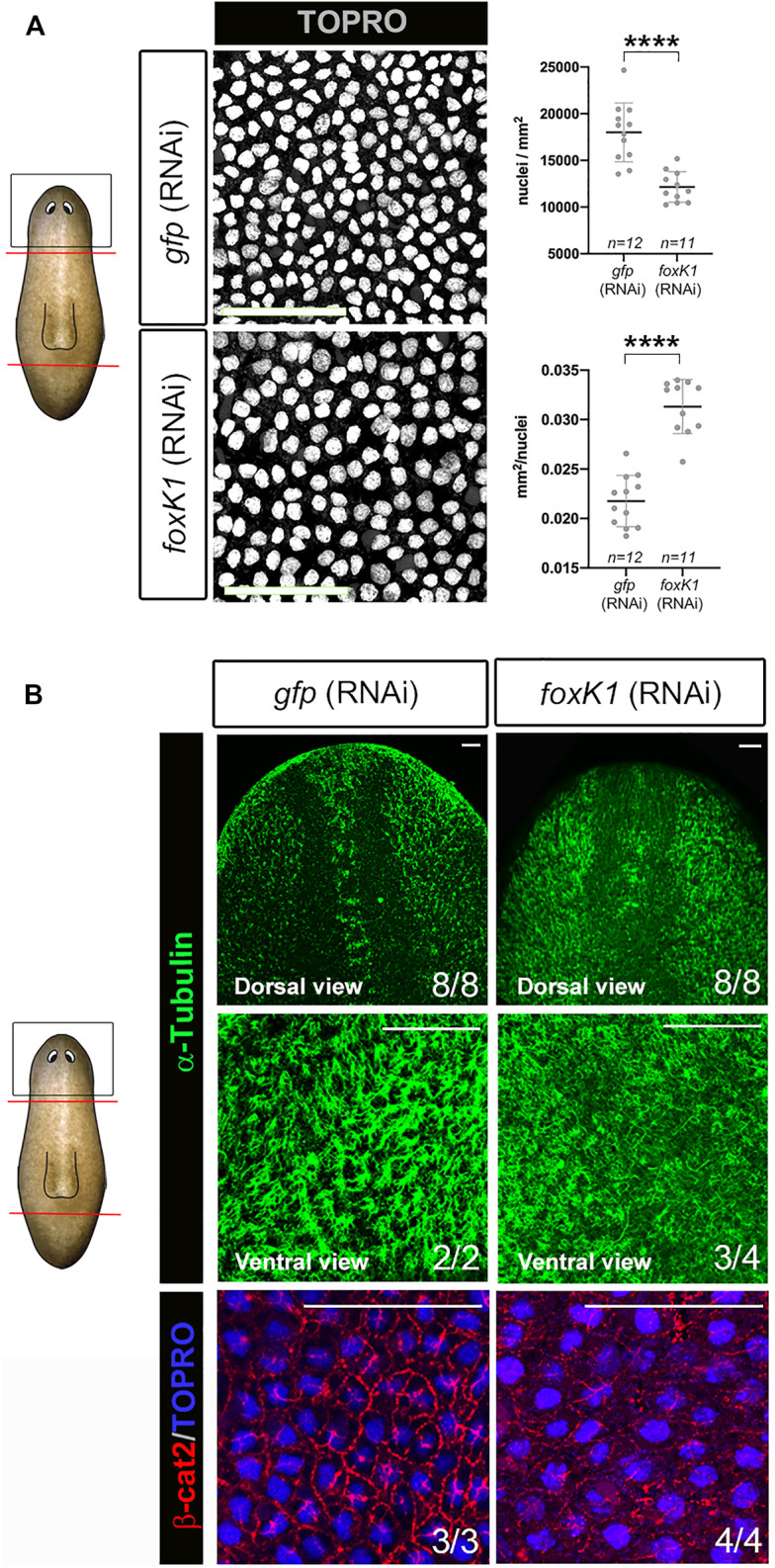
*foxK1* silenced planarians show defects in the epidermis. **(A)** TO-PRO®-3 staining of the epidermis in anterior wounds and quantification showing that the epidermal nuclear density decreases (number of nuclei/mm2 of the area of the confocal section) while the size for each nucleus increases (average size in mm2 of each nucleus in the confocal section of every individual) after *foxK1* silencing. Animals at 7 days of regeneration are shown. All images are dorsal views. Values represent the mean ± standard deviation [s.d] of a mean of at least 11 planarians per condition. Scale bar: 50 µm ****p-value < 0.0001. **(B)** Immunostaining of the epidermis in anterior wounds. Anti-*α*-tubulin shows the aberrant regeneration of cilia along the dorsal midline and the ventral epidermis. Merge of the immunostaining of anti-*β*-catenin2 and TO-PRO®-3 in the dorsal region between the eyes showing a misorganization of the epithelial adherens junctions. Animals at 7 days of regeneration are shown. Scale bar: 50 µm. In the schematic planarians, red lines indicate the amputation planes and black squares the region analysed.

*foxK1* appears expressed in the neural and in the epidermal lineages according to the published single-cell RNAseq data (Supplementary Figure S1C). Furthermore, its inhibition produced epidermal lesions (Figure 1A). Thus, we analyzed deeper the integrity of the epidermal tissue in RNAi animals. Quantification of the nucleus demonstrated a decrease of cell density in the epidermis (Figure 3A). Interestingly, the nucleus appeared bigger in *foxK1* RNAi animals, as demonstrated by the quantification of their area (Figure 3A). Furthermore, the cilia of the epidermal cells in the dorsal midline were not properly regenerated (Figure 3B) and, in the ventral part, appeared disorganized (Figure 3B). Immunolabeling of adherens junctions with anti-β-catenin2 antibody indicated that these structures were not properly assembled in *foxK1* silenced animals, since the signal was dimmer and the pattern was irregular (Figure 3B).

On the other hand, other tissues such as the digestive or the excretory systems seemed not to be affected after *foxK1* inhibition (Supplementary Figures S2B–D).

Overall, these results suggest that *foxK1* is required for maintenance and regeneration of the epidermis in planarians.

### Apoptotic Response is Affected in *foxK1* RNAi Animals

**FIGURE 4:**
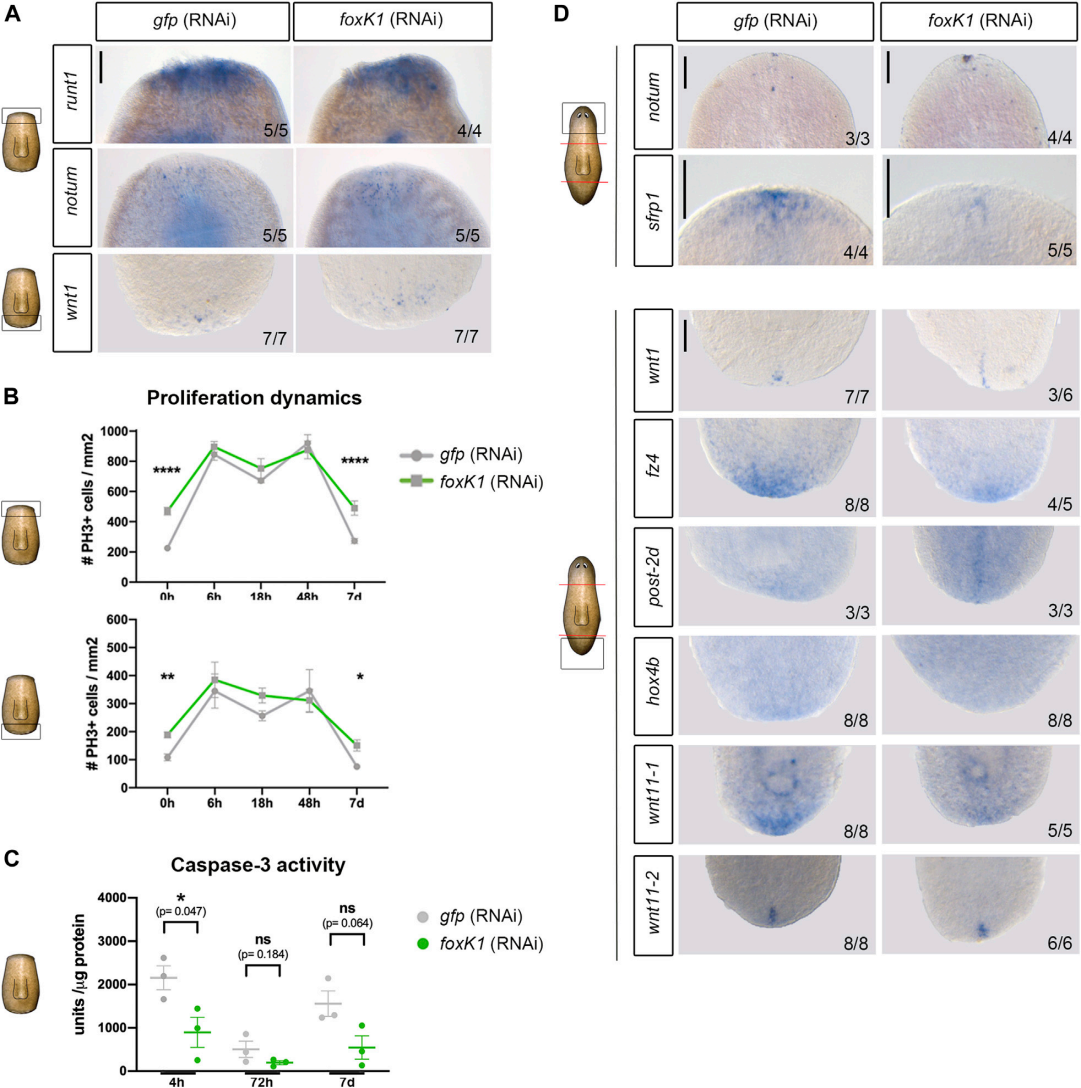
Apoptotic response is affected in foxK1 RNAi animals, but the polarity establishment and proliferative response occurs normally. **(A)** Whole-mount *in situ* hybridization analysis of the expression of the early wound response genes in controls and *foxK1* RNAi animals 6 h post amputation (*runt1* and *notum*) and 12 h post amputation (*wnt1*). From top to bottom: *runt1* in anterior wounds, *notum* in anterior wounds and *wnt1* in posterior wounds. Scale bar: 100 µm. **(B)** Quantification of mitotic PH3^+^ immunolabeled cells after silencing *foxK1* at several time points after anterior-posterior amputation. h, hours; d, days (*p-value < 0.05; ** *p*-value < 0.01 ****p-value < 0.0001. Student’s *t*-test). Values represent the mean ± standard error of the mean [s.e.m] of a mean of at least 4 samples per time point and condition. **(C)** Quantification of caspase-3 activity after silencing *foxK1* at several time points after anterior-posterior amputation. h, hours; d, days (*p-value < 0.05; Student’s *t*-test). Values represent the mean ± standard error of the mean [s.e.m] of a mean of 3 biological samples per time point and condition. **(D)** Whole-mount *in situ* hybridization analysis of the expression of the genes responsible for establishing the anterior and posterior identity in controls and *foxK1* RNAi animals 3 days post amputation *(notum, wnt1, frz4, post2d* and *hox4b*), 6 days post amputation (*wnt11_1* and *wnt11_2*) and 7 days post amputation (*sfrp1*). Anterior polarity genes (from top to bottom): *notum* and *srfp1*. Posterior polarity genes (from top to bottom): *wnt1, frz4, post-2d, hox4b, wnt11-1 and wnt11-2*. Scale bar: 100 µm. In the schematic planarians, red lines indicate the amputation planes and black squares the region analysed.

Regeneration of the missing tissues in planarians is preceded by the triggering of early signals that activate the expression of the early genes as well as the proliferative and apoptotic response (Saló and Baguñà 1984; Wenemoser and Reddien 2010; Wenemoser et al., 2012; Pirotte et al., 2015; Owlarn et al., 2017; Jaenen et al., 2021). Analysis of the expression of the early response gene *runt1*, as well as the polarity genes *notum* and *wnt1*, whose early expression is independent of polarity and occurs after any injury (Wenemoser et al., 2012; Sandmann et al., 2011; Petersen and Reddien 2009, 2011), indicated that the early gene response was not affected after *foxK1* inhibition (Figure 4A).

In addition, quantification of the proliferative response near the anterior and the posterior wounds showed that the dynamics of proliferation was not perturbed in *foxK1* RNAi animals, and the two mitotic peaks at 6 and 48 h described during planarian regeneration occurred normally (Figure 4B). It must be noted, however, that proliferation appeared to be increased in regenerating animals before amputation, but this did not interfere with a proper proliferative response after injury. On the other side, quantification of the apoptotic response showed a decrease in *foxK1* RNAi animals, both at early (4 h) and late phases of regeneration (7 days) (Figure 4C).

These results indicate the only event found deregulated in *foxK1* RNAi animals during the early wound response is the activation of apoptosis.

### *foxK1* RNAi Animals Show a Correct Polarity Establishment

During planarian regeneration, the general early wound response is followed by a pole-specific response, which includes the expression of genes specific to anterior or posterior wounds that are required to specify the identity and to differentiate the proper missing tissue (Wurtzel et al., 2015; Cebrià, Adell, and Saló 2018). To clarify whether the problems of *foxK1* RNAi planarian regeneration could be associated with an incorrect pole-specific response, we analyzed several genes specific to anterior and posterior regeneration. The results showed that both anterior and posterior poles were properly formed in *foxK1* silenced planarians, since *notum* and *wnt1* were restricted to anterior and posterior wounds, respectively (Figure 4D) (Petersen and Reddien 2011; Adell et al., 2009). *post2-d* and *wnt11-2*, two positional control genes (PCG) (Witchley et al., 2013) that are specifically expressed in posterior facing wounds after *wnt1* (Adell et al., 2009; Currie et al., 2016; Tewari et al., 2019), also showed a proper expression in *foxK1* RNAi planarians (Figure 4D). However, the expression of some anterior and posterior PCG appeared decreased in *foxK1* RNAi animals, such as *sfrp1* in anterior; and *fz4*, *hox4b* and *wnt11-1* in posterior (Gurley, Rink, and Alvarado 2008; Petersen and Reddien 2008; Currie et al., 2016).

These results suggest that polarity was not affected in *foxK1* silenced planarians, even though the inability to regenerate a correct head and tail was associated with a decreased expression of some markers of anterior and posterior polarity.

## Discussion

In this study, we have found that inhibition of *foxK1* impedes correct regeneration of missing structures, while inhibition of *foxK2* or *foxK3* only produces mild defects. The strong phenotype observed after *foxK1*, but not *foxK2* or *foxK3* inhibition, could be due to the fact that the last two are mainly expressed in differentiated cells (Pascual-Carreras et al., 2021), while *foxK1* is also expressed in progenitors and in neoblasts. *foxk1* RNAi animals cannot properly regenerate the nervous system or the epidermis, which correlates with a decrease in the number of differentiated neural and epidermal cells. At least in the case of the nervous system, *foxK1* RNAi animals also show a decrease in the number of progenitors (*sim+/*PIWI1+) cells. However, the number of cycling cells is not decreased. This result could indicate that *foxK1* is required for maintenance and differentiation of the neural precursors and may be for specification of neoblasts towards the neural fate. This result agrees with a recent study in human ESCs, in which the FoxK2 transcription factor is found to be bound to thousands of regulatory regions and to act as a pioneer factor, playing a role in enhancer priming during differentiation (Ji et al., 2021). Thus, *foxK1* could also act as a pioneer factor during neural determination in planarians. This pioneer role has been already demonstrated for other Fox families, such as FoxA, FoxD or FoxH (Krishnakumar et al., 2016; Charney et al., 2017). In human ESCs, FoxK2 binding to enhancers is maintained as cells differentiate to neural precursor cell types (Ji et al., 2021). This could also be the case of *foxK1* in planarians. In contrast to other Fox factors, *foxK1* could be recruited not only in enhancer regions prior to their activation, but its binding could be retained as enhancers become activated during differentiation, since both progenitor and differentiated cells are decreased in RNAi animals.

Although the cephalic ganglia are more affected than VNCs in *foxK1* RNAi planarians, *foxK1* seems to be required for nervous system differentiation in general, since posterior fascicles of the VNCs appear thinner and the decrease in the expression of *sim* affects to all the body regions.

The defect in the eyes of *foxK1* RNAi animals is one of the first traits that can be observed, since they are the only structures clearly identified when planarians regenerate the head. With the analysis of specific markers, we demonstrate that eyes are not properly specified nor differentiated, since both eye precursor and eye differentiated cells are decreased. However, we cannot clarify whether this defect is due to an autonomous role of *foxK1* in the eye cells or it is linked to the inability of these animals to regenerate the cephalic ganglia, since the eyes connect to the brain while they both are regenerating. We have not observed the expression of *foxK1* in eye cells, but we cannot discard it.

*FoxK1* RNAi animals die after developing epidermal lesions, thus it can be due to its role in the maintenance of the epidermal layer. We observed a decrease in epidermal cell density and an increase in the size of the nucleus, which agrees with the role of *foxK1* for correct terminal differentiation of epidermal cells. However, we cannot conclude whether *foxK1* is also required for maintenance and differentiation of epidermal progenitors, and whether it also acts as a pioneer factor in the epidermal lineage, since the number of epidermal precursors has not been analyzed. It is tempting to speculate that the increase in the size of the nucleus could be related with a decompaction of the chromatin, which would agree with the role of *foxK*1 as a pioneer factor, although it could also be due to other mechanisms.

*FoxK1* expression is not neural or epidermal specific, thus it could be also required for specification or differentiation of other tissues. However, its RNAi inhibition apparently only affects the ectodermal lineages, as the digestive or the excretory system appear normal.

Despite in other models, as *Drosophila*, *foxK* has been involved in the control of proliferation of myogenic stem cells (Garry et al., 2000), our results indicate that in a stem cell-based model as planarians the FoxK family is not required for neoblast proliferation. *foxK2* and *foxK3* are not expressed in neoblasts, and *foxK1* is expressed in neoblasts but we have found that the bimodal proliferative response triggered by injury is correct in *foxK1* RNAi planarians, and in animals that have already regenerated there are more proliferative cells. This result agrees with the hypothesis that *foxK1* is activating neoblast and progenitor enhancers required for determination and differentiation, but it is not required for their proliferation. If *foxK1* is required to prime neoblasts, they would maintain their naive and cycling properties when *foxK1* is not functional, which would explain the increase in proliferation seen in regenerated animals that must maintain their tissues. However, the triggering of stem cell proliferation after injury is not dependent on *foxK1*.

*foxK1* RNAi animals present a correct early mitotic response and early wound gene expression. However, we have found that inhibition of *foxK1* decreases the early apoptotic response at 4 h of regeneration, suggesting that this gene could have a key role in controlling apoptosis. In this study we have not been able to clarify the impact of the early apoptotic decrease in the phenotype observed. However, the fact that the apoptotic activity seems also reduced at later stages of regeneration in *foxK1* RNAi planarians suggests that the observed decrease in the number of neural progenitors and epidermal cells is not due to an increase in their cell death.

Polarity establishment is neither affected in *foxK1* RNAi planarians. Some PCGs are not correctly expressed, such as *sfrp1* in anterior and *fz4*, *hox4b* and *wnt11-1* in posterior. However, this could be associated with the inability to form a proper head or tail, but not to the incorrect pole formation, since *notum* and *wnt1* are correctly expressed at all regeneration stages. A possibility is that the expression of those PCGs genes could require the differentiation of a nervous system.

While several Fox proteins have been implicated in enhancer activation in a tissue-specific manner, the role of the FoxK has been associated to a range of biological processes apparently unconnected. This could be mainly due to its ubiquitous expression and to having an additional conserved domain, the FHA domain, which binds to multiple proteins. Analysing *foxK1* function in planarians we have been able to characterize a specific role of *foxK1* in the differentiation of the neural tissue, possibly by acting as a pioneer factor, as other members of the Fox family, which allows and maintains the activation of enhancers during differentiation of neural progenitors. Furthermore, the role of *foxK1* in controlling apoptosis deserves further attention, since a functional relationship between FoxK and apoptosis has only been suggested in cancer cells (Liu et al., 2019).

## Materials and Methods

### Animal Maintenance

*S. mediterranea* from the asexual clonal line BCN-10 were used for all experiments. Animals were maintained at 20°C in 1X PAM water as previously described (Cebrià et al., 2007). Animals were fed twice per week with organic veal liver and were starved for at least 1 week before experiments. For irradiation experiments, planarians were exposed to 86 Gray (Gy) of γ-irradiation.

### RNA Interference

Double-stranded RNA (dsRNA) for *Smed-foxK* genes were synthesised as previously described (Sánchez Alvarado and Newmark 1999). The region amplified by the *foxK1* specific forward primer 5′ AAT​CTC​ATT​CTT​TTA​TTC​CT 3′ and reverse primers 5′ AGT​ATC​GTT​CAT​CAG​TCC​AT 3′ or 5′ TTG​AGC​GTA​TGA​ATA​TGG​AGG 3’ was used to synthesize the dsRNA. The injection protocol consisted of 1 round of 4 consecutive days of injections or 2 rounds of 3 consecutive days of injections separated by a 4-days interval. A Nanoject II (Drummond Scientific, Broomall, PA, United States) was used to administer 3 injections of 32 nl of dsRNA (1 μg/μl) per day. Control animals were injected with *gfp* dsRNA*.* In each round, 1 day after the last injection, planarians were amputated to induce anterior and posterior regeneration.

### Single-Cell Sequencing Data

*FoxK1,2,3* genes expression profile (dd_Smed_v6_4500, dd_Smed_v6_5767 and dd_Smed_v6_7583, respectively) were obtained from the planaria single-cell database hosted by the Rajewsky lab at the Berlin Institute for Medical Systems Biology of the Max Delbrück Center, Berlin (Plass et al., 2018) and the planaria single-cell database hosted by the Sánchez Alvarado lab at Planosphere website (Zeng et al., 2018).

### Whole-Mount *In Situ* Hybridisation and Whole-Mount Fluorescent *In Situ* Hybridisation

Whole-mount *in situ* hybridisation (WISH) (Currie et al., 2016) whole-mount fluorescent *in situ* hybridisation (WFISH) (King and Newmark 2013) were performed as previously described. Riboprobes for *in situ* hybridisation were synthesised using the DIG RNA labe ling kit (Sp6/T7, Roche) following the manufacturer’s instructions. Samples were mounted in 70% glycerol/PBS solution.

### Immunohistochemistry

Whole-mount immunohistochemistry was performed as previously described (Ross et al., 2015). Treated animals were euthanised by immersion in cold 2% HCl in ultrapure H_2_O for 5 min, washed with PBS-Tx (PBS +0.3% Triton X-100) at room temperature (RT), and placed in a fixative solution (4% formaldehyde in PBS-Tx) for 15 min at RT with agitation. Subsequently, samples were washed with PBS-Tx and bleached in 6% H_2_O_2_ (in PBS-Tx) at RT for 16 h under direct light. Bleached animals were then washed with PBS-Tx, incubated for 2 h in 1% blocking solution (1% BSA in PBS-Tx), and overnight at 4°C in the primary antibody (diluted in blocking solution). The following primary antibodies were used: anti-phospho-histone3 (PH3, Cell Signaling Technology) to detect mitotic cells, diluted 1:300; anti-SYNAPSIN, a pan-neural marker (anti-SYNORF1, Developmental Studies Hybridoma Bank), diluted 1:50; anti-VC-1, specific for planarian photosensitive cells (Sakai et al., 2000), diluted 1:15,000; anti-SMEDWI-1, specific for neoblasts, diluted 1:1,500 (Rouhana et al., 2010) (März, Seebeck, and Bartscherer 2013); polyclonal antibody against Smed-*β*-catenin-2 used to visualize adherens junctions (Chai et al., 2010), and used at a 1:2,000 dilution; the AA4.3 antibody (Developmental Studies Hybridoma Bank), specific for α-tubulin to visualize the epithelial cilia, diluted 1:20. The following secondary antibodies were used: Alexa-488-conjugated goat anti-mouse (Molecular Probes), diluted 1:400; and Alexa 568-conjugated goat anti-rabbit (Molecular Probes), diluted 1:1,000. Samples were mounted in 70% glycerol/PBS solution. Nuclei were stained with DAPI (1:5000; Sigma-Aldrich) and TO-PRO®-3 (1:3,000, Thermo Fisher Scientific, Waltham, MA, United States).

### Phototactic Assay

Phototactic assay was carried out using a modified version of the method described by (Inoue et al., 2004). Planarian behavior was recorded during 120 s using an overhead digital video camera (Canon EOS550D). The behavior analysis software SMART v.2.5.21 (Panlab, Spain) was used to quantify the time spent by the animals in each of the three virtual subdivisions of a transparent container of 60 × 30 × 10 mm, filled with planarian water. To obtain a light gradient, the container was protected by a black screen with a hole that allowed the entrance of 500 lux of white light from one side of the container.

### Caspase-3 Activity Assay

The protein concentration of the cell lysates of three biological samples of 4–7 planarians each was measured using BioRad protein reagent. Fluorometric analysis of caspase-3 activity was performed as described previously (González-Estévez et al., 2007) using 16 μg of protein extract after incubation for 2 h at 37°C with the caspase-3 substrate Ac-DEVD-AMC. A Fluostar Optima microplate fluorescence reader (BMG Labtech) and a luminescence spectrophotometer (Perkin- Elmer LS- 50) were used to measure luminescence. The following settings were applied: excitation, 380 nm; emission, 440 nm. Three technical replicates were analysed per condition.

### Microscopy, Image Acquisition, and Image Analysis

Live animals were photographed with an sCM EX-3 high end digital microscope camera (DC.3000s, Visual Inspection Technology). WISH, WFISH, and immunostained animals were observed with a Leica MZ16F stereomicroscope. Images were captured with the ProGres C3 camera (Jenoptik) and then processed in Photoshop CS6 for publication. Representative images of WFISH and immunostained animals were captured with confocal laser scanning microscopy (Leica TCS-SPE microscope) and processed in ImageJ2.3.0 and Photoshop CS6 for publication.

### Statistical Analysis

All comparisons were performed using the unpaired Student’s t-test, after first confirming data normality and homogeneity using the Shapiro-Wilk test.

## Data Availability

The original contributions presented in the study are included in the article/Supplementary Material, further inquiries can be directed to the corresponding author.

## Supplementary Material

Supplementary Figure S1 RNAi silencing and expression pattern of the three *foxK S. mediterranea* paralogs. **(A)** *foxK2* or *foxK3* silenced planarians regenerate the missing head and tail. Scale bar: 500 µm. **(B)** Phototactic assay. Schematic representation of the position of treated planarians in each of the three subdivisions of 20 cm each after 120 s of light exposure. Red dots represent the position of *foxK1* silenced animals and blue dots the position of *gfp* RNAi control planarians. Graphical representation of the quantification of the velocity (meter/seconds) of *gfp* and *foxK1* silenced planarians showing the reduced mobility of *foxK1* silenced planarians. **(C)** Whole-mount in situ hybridization of *foxK1* in intact, γ-irradiated and planarians at 2 days of regeneration after anterior-posterior amputation. Scale bar: 200 µm. **(D)** Schematic representation of single-cell transcriptomic expression of *foxK* genes [according to (Plass et al., 2018)]. It must be noted that in a previous publication (Pascual-Carreras et al., 2021), these plots were not properly assigned, but this is corrected in the present publication. **(E)** Schematic representation of single-cell transcriptomic expression of *foxK1* in all planarian neoblasts (Nb) subtypes [according to (Zeng et al., 2018)]. **(F)** Relative gene expression of *foxK1* and *piwi1* for the individual stages during differentiation of the epidermal and neuronal planarian cell lineages (in pseudotime) according to single cell analysis [https://shiny.mdc-berlin.de/psca/, (Plass et al., 2018)]. a.u, arbitrary units. In the schematic planarians, red lines indicate the amputation planes and black squares the region analysed.

Supplementary Figure S2 Expression pattern of tissue-specific markers in *foxK1* RNAi planarians. **(A**) Whole-mount in situ hybridization of the neural marker *sim* (Cowles et al., 2013) reveals a decreased number of sim-expressing cells all along the planarian body of *foxK1* silenced planarians. Animals at 7 days of regeneration are shown. Scale bar: 100 µm. **(B)** Whole-mount in situ hybridization of *innexin10* (Nogi and Levin 2005) labeling the planarian excretory system reveals correct regeneration of excretory cells in *foxK1* silenced planarians. Animals at 7 days of regeneration are shown. Scale bar: 100 µm. **(C)** Whole-mount in situ hybridization of the gut marker *pk* (Fraguas et al., 2014) showing that treated planarians regenerate correctly the gut. Whole-mount immunostaining against *α*-tubulin showing a detail of the newly regenerated posterior gut branches. Animals at 7 days of regeneration are shown. Scale bars: 100 µm.
