# Supplementary figures and images for: FoxK1 is Required for Ectodermal Cell Differentiation During Planarian Regeneration
Source: Front Cell Dev Biol. 2022 Feb 22;10:808045. doi: 10.3389/fcell.2022.808045 (PMC8901602; doi:10.3389/fcell.2022.808045)

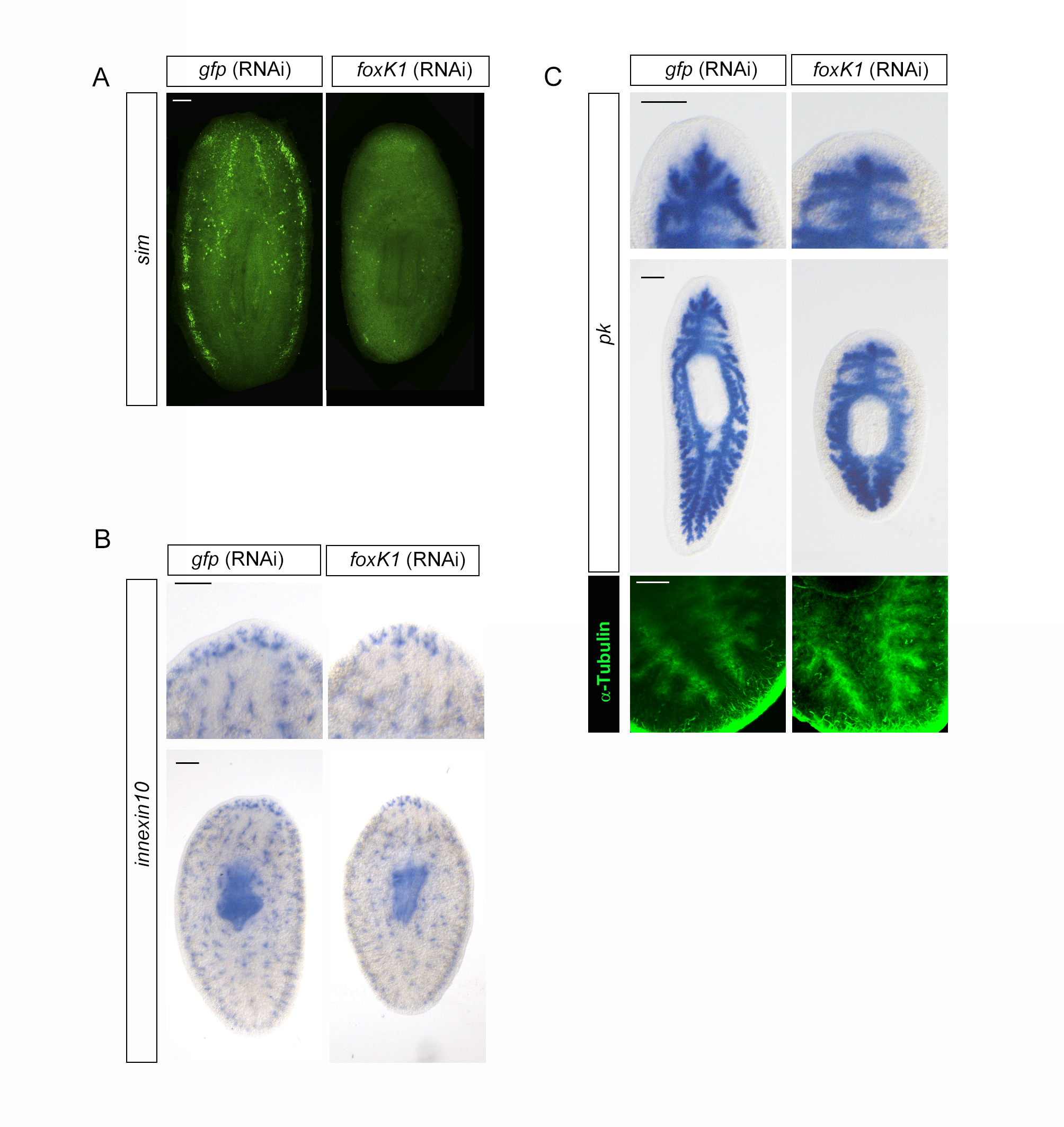

Supplement: Supplementary file 1 [file Image2.TIF]

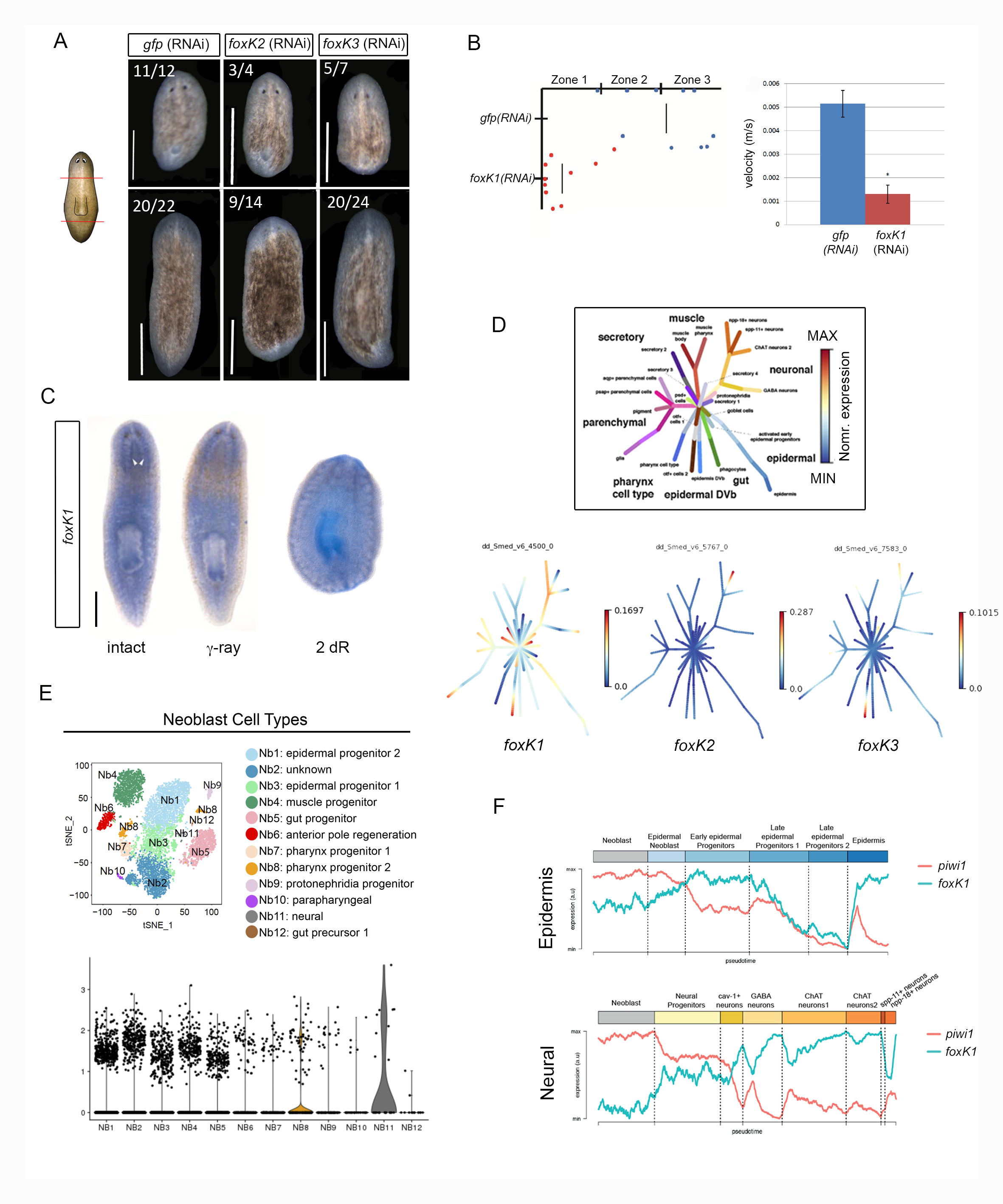

Supplement: Supplementary file 2 [file Image1.TIF]
